# Endoscopic submucosal dissection for adenoma in gastric adenocarcinoma and proximal polyposis of the stomach

**DOI:** 10.1055/a-2119-0677

**Published:** 2023-07-27

**Authors:** Yuri Saito, Mitsunori Kusuhara, Akiko Ohno, Naohiko Miyamoto, Yu Hada, Junji Shibahara, Tadakazu Hisamatsu

**Affiliations:** 1 Department of Resident Centers, Kyorin University School of Medicine, Tokyo, Japan; 2 Department of Gastroenterology and Hepatology, Kyorin University School of Medicine, Tokyo, Japan; 3 Department of Pathology, Kyorin University School of Medicine, Tokyo, Japan

Gastric adenocarcinoma and proximal polyposis of the stomach (GAPPS) is an autosomal-dominant syndrome developing gastric carcinoma with a background of fundic gland polyposis 1 . Although prophylactic total gastrectomy is considered given the high incidence of gastric cancer 2 3 , there is no consensus/guideline. We report a case of treating a pyloric gland adenoma (PGA) in GAPPS with endoscopic submucosal dissection (ESD).

A 54-year-old woman was referred to our hospital with a diagnosis of fundic gland polyps (FGPs). FGPs were localized in the gastric body and fundus ( Fig. 1 ), but there were no polyps in the antrum and duodenum. A 20-mm white elevated lesion was observed in the greater curvature of the upper body. Under narrow-band-imaging magnifying endoscopy, arcuate glandular duct structures were observed, and the demarcation line could be identified ( Fig. 2 ). She had several relatives with gastric cancer; particularly her brother was diagnosed with fundic gland polyposis. Hence, we suspected a gastric-type tumor associated with GAPPS.

**Fig. 1:**
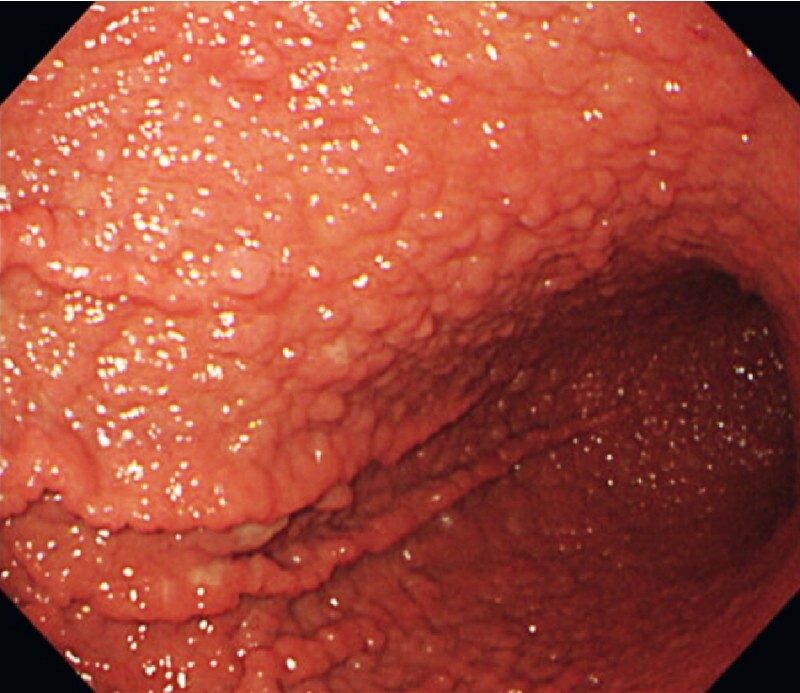
Endoscopic findings of the stomach (distant view). Fundic gland polyps were observed in the gastric body and fundus, except for the lesser curvature.

**Fig. 2:**
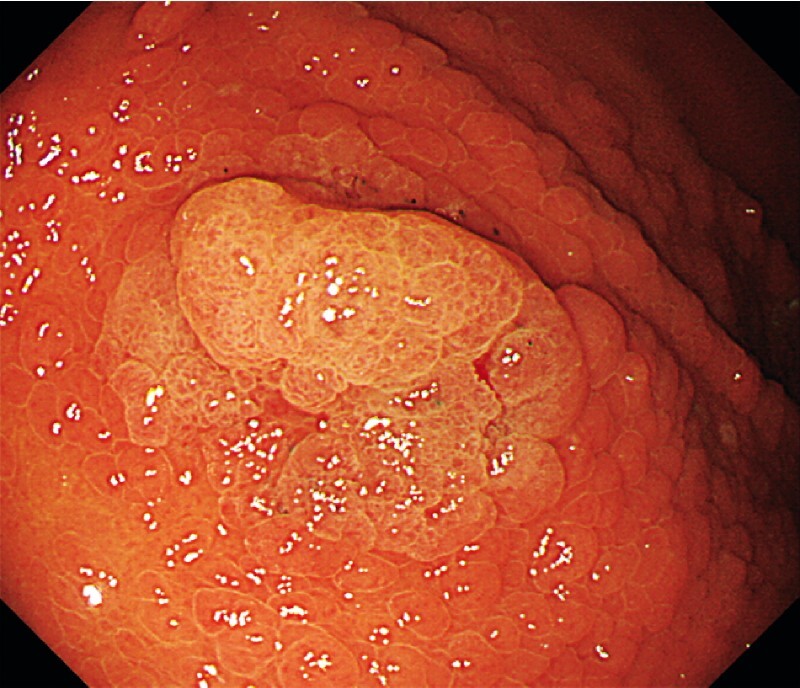
Endoscopic findings of the tumor. A 20-mm white elevated lesion was observed in the greater curvature of the upper body.

ESD was performed because the patient refused the surgery ( Video 1 ). The lesion was resected en bloc using the clip-and-thread traction method ( Fig. 3 ). The lesion consisted of closely packed neoplastic glands resembling pyloric glands. The tumor exhibited pronounced cytological and architectural atypia in some areas ( Fig. 4 ). Immunohistochemistry revealed diffuse reactivity to MUC6 and focal reactivity to MUC5AC. The histological diagnosis was PGA with high grade dysplasia.

**Video 1** A case of treating a pyloric gland adenoma in gastric adenocarcinoma and proximal polyposis of the stomach with endoscopic submucosal dissection.


**Fig. 3:**
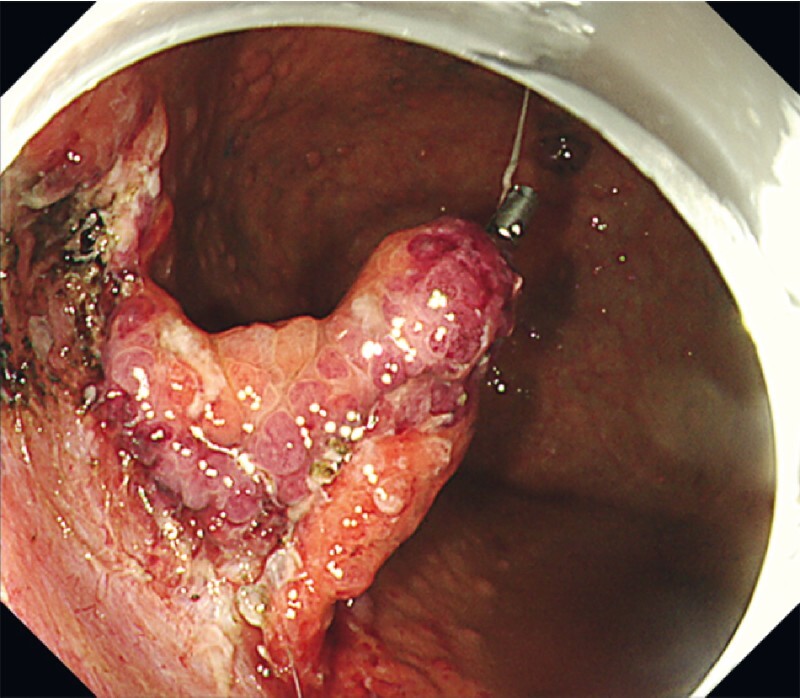
Procedure of endoscopic submucosal dissection. The lesion was resected en bloc by the clip-and- thread traction method.

**Fig. 4:**
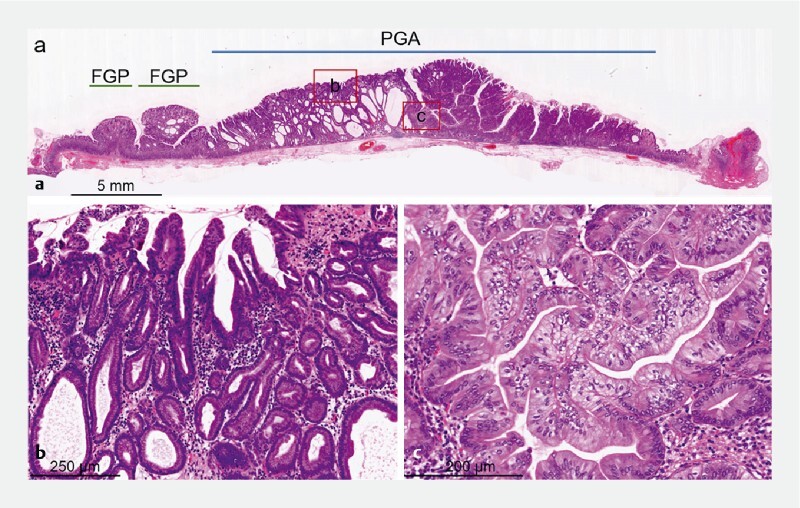
Histopathological findings (hematoxylin and eosin staining). **a** The main lesion was a pyloric gland adenoma (PGA) showing a flat elevation. Two fundic gland polyps were adjacent to PGA. **b** The PGA was composed of pyloric-type glands. **c** High grade atypia was noted.

Genome analysis of the *APC* gene using peripheral blood demonstrated a point mutation c.-191T > C in exon 1B, a characteristic mutation of GAPPS 4 . The final diagnosis was pyloric gland adenoma associated with gastric adenocarcinoma and proximal polyposis of the stomach.

Although the effectiveness of endoscopic surveillance is unestablished 2 , considering the high risk of carcinogenesis in the residual stomach, close endoscopic follow-up is planned. It is interesting that a white patch in fundic gland polyps as observed in this case is associated with a high rate of proximal gastric cancer in familial polyposis 5 .

Endoscopy_UCTN_Code_CCL_1AB_2AD_3AB

## Notes

Endoscopy E-Videos

https://eref.thieme.de/e-videos
*E-Videos* is an open access online section of the journal *Endoscopy* , reporting on interesting cases and new techniques in gastroenterological endoscopy. All papers include a high-quality video and are published with a Creative Commons CC-BY license. Endoscopy E-Videos qualify for HINARI discounts and waivers and eligibility is automatically checked during the submission process. We grant 100% waivers to articles whose corresponding authors are based in Group A countries and 50% waivers to those who are based in Group B countries as classified by Research4Life (see: https://www.research4life.org/access/eligibility/ ). This section has its own submission website at https://mc.manuscriptcentral.com/e-videos

## References

[1] Worthley D L Phillips K D Wayte N Gastric adenocarcinoma and proximal polyposis of the stomach (GAPPS): a new autosomal dominant syndrome Gut 2012 61 774 779 21813476 10.1136/gutjnl-2011-300348

[2] Repak R Kohoutova D Podhola M The first European family with gastric adenocarcinoma and proximal polyposis of the stomach: case report and review of the literature Gastrointest Endosc 2016 84 718 725 27343414 10.1016/j.gie.2016.06.023

[3] Rudloff U Gastric adenocarcinoma and proximal polyposis of the stomach: diagnosis and clinical perspectives Clin Exp Gastroenterol 2018 11 447 459 30584346 10.2147/CEG.S163227 PMC6284852

[4] Li J Woods S L Healey S Point mutations in exon 1B of APC reveal gastric adenocarcinoma and proximal polyposis of the stomach as a familial adenomatous polyposis variant Am J Hum Genet 2016 98 830 842 27087319 10.1016/j.ajhg.2016.03.001 PMC4863475

[5] Kunnathu N D Mankaney G N Leone P J Worrisome endoscopic feature in the stomach of patients with familial adenomatous polyposis: the proximal white mucosal patch Gastrointest Endosc 2018 88 569 570 30115306 10.1016/j.gie.2018.04.010

